# Nutrition Knowledge and Eating Habits of Healthcare Workers at a Tertiary Hospital in Limpopo Province, South Africa

**DOI:** 10.3390/ijerph22121838

**Published:** 2025-12-09

**Authors:** Sindzile Pearl Khosa, Morentho Cornelia Phetla, Mashudu Manafe

**Affiliations:** Department of Human Nutrition & Dietetics, Sefako Makgatho Health Sciences University, Pretoria 0001, South Africa; khosacp@gmail.com (S.P.K.); mashudu.manafe@smu.ac.za (M.M.)

**Keywords:** eating habits, healthcare workers, healthy eating, nutrition knowledge

## Abstract

Poor nutrition knowledge and unhealthy eating habits are major contributors to the global rise in non-communicable diseases. Despite the abundance of nutrition information, many individuals struggle with adopting and maintaining healthy eating patterns. This study assessed the association between nutrition knowledge and eating habits among healthcare workers (HCWs) at a tertiary hospital in the Limpopo province of South Africa. A cross-sectional, descriptive study was conducted among 303 healthcare workers. The data collection period was from the 18 of April to the 5 of May in 2023. Data were collected using a self-administered questionnaire consisting of three sections: demographic information, nutrition knowledge, and eating habits. Descriptive and inferential statistics were applied, with a significance level set at *p* ≤ 0.05. More than half of the participants (n = 165; 55%) demonstrated a moderate level of nutrition knowledge (scores of 60–79%), while 113 (37%) had a high level of knowledge (80–100%) and 25 (8%) had a low level of knowledge (<60%). Only 16% of participants reported healthy eating habits, while 84% reported unhealthy eating habits. A statistically significant association was observed between occupation and nutrition knowledge (*p* < 0.05), with medical doctors showing higher knowledge levels than nurses and allied health professionals. No significant association was found between nutrition knowledge and eating habits (*p* > 0.05). Notably, even participants with high nutrition knowledge did not consistently report healthy eating behaviours. Although most healthcare workers possessed moderate to high nutrition knowledge, this was not consistently reflected in their eating practices. Future research should explore factors influencing the gap between nutrition knowledge and eating behaviour among healthcare professionals.

## 1. Introduction

Poor nutrition knowledge and unhealthy eating habits are major contributors to the global rise in non-communicable diseases (NCDs) such as obesity, cardiovascular diseases, Type 2 diabetes mellitus, and some cancers [1,2]. Despite the abundance of nutrition information, many individuals continue to struggle with adopting and maintaining healthy eating patterns. Research has shown that while nutrition knowledge can positively influence dietary behaviour, this knowledge alone often does not result in healthier eating [3,4,5].

In sub-Saharan Africa, the burden of non-communicable diseases is increasing rapidly, partly due to poor diet quality and lifestyle changes associated with urbanisation and socio-economic transitions [6]. In South Africa specifically, individuals often face the dual burden of malnutrition and overnutrition, leading to overweight and obesity. The role of nutrition education in solving these problems is increasingly recognised, while the implementation and transformation of behaviour remain stagnant [7].

In South Africa, concerns about the prevalence of overweight and obesity are increasing, mainly due to unhealthy food choices and sedentary lifestyles [8,9]. The country is experiencing a nutritional transition characterised by an increase in energy-intensive, nutrient-poor food consumption and a decrease in physical activity levels [10]. South Africans face a nutritional transition due to urbanisation (increased access to fast foods and take-away convenience) and a transition from traditional meals to westernised diets, which are highly processed foods with high fat and sugar [11]. Poor eating habits are associated with a high prevalence of NCDs, stress on the health system, and a reduction in productivity among the working population [12].

Healthcare workers (HCWs), who are expected to promote and model healthy eating habits, are not exempt from these challenges. Several studies have shown that HCWs, despite being health professionals, often engage in poor eating practices and may lack sufficient nutrition knowledge [13,14]. Although HCWs typically acquire nutrition knowledge during their training, many struggle to translate this into practice due to competing priorities and work-related stressors [15]. The demands of the profession, long hours, and high patient loads, often lead HCWs to neglect their well-being [16].

Importantly, HCWs who maintain a healthy lifestyle are more likely to promote healthy eating to patients [17]. On the contrary, HCWs who do not give priority to their own health may be less inclined or confident to engage in nutritional counselling. Nutritional knowledge has also been shown to influence the choice of food and healthcare practices of HCWs, but knowledge alone may not be sufficient to change habits [18]. In South Africa, studies have shown that some healthcare workers, especially women, misclassify their own weight, a factor that may contribute to underestimating the health risks associated with a poor diet [12]. Beliefs about the body are influenced by cultural beliefs and socio-economic status in some communities. It is often believed that people who are overweight and obese are healthy, happy, married, and wealthy, and that those who are underweight and healthy (25 kg/m^2^) are considered to be poor and ill with diseases such as human immunodeficiency virus (HIV), tuberculosis, and cancer [18]. These concepts lead to them being considered healthy when they are actually overweight or obese.

Given the central role of HCWs in health promotion and disease prevention, it is crucial to understand their level of nutrition knowledge and eating habits. Although nutrition knowledge is considered a key determinant of healthy eating, the relationship between nutrition knowledge and healthy eating remains unclear. According to the health belief model (HBM), participation in health-promoting behaviours such as healthy eating depends not only on the knowledge of individuals, but also on their perception of sensitivity to disease, the severity of perceived health results, the benefits and barriers perceived to action, and self-efficacy. Thus, even when medical workers have sufficient knowledge of nutrition, competing demands such as heavy workloads, time constraints, and the availability of unhealthy food options, can act as perceived obstacles to healthy eating behaviours [19]. This study aims to assess the nutrition knowledge and eating habits of healthcare workers (HCWs) at a tertiary hospital in the Limpopo province of South Africa

## 2. Methods

### 2.1. Study Design

The study used a cross-sectional descriptive design to describe the knowledge and eating habits of the participants.

### 2.2. Study Site

The study was conducted at a tertiary hospital in the Limpopo province of South Africa. The hospital is situated in a semi-urban area with commercial facilities, including malls, shopping centres, and various restaurants. Additionally, food vendors that sell fast food, fruits, and vegetables are located outside the hospital, as well as a tuck shop within the hospital’s premises.

### 2.3. Study Population

The target population comprised healthcare workers employed at a tertiary hospital, including nurses, medical doctors, and allied health professionals such as dietitians, physiotherapists, speech therapists, audiologists, dentists, dental therapists, radiographers, social workers, podiatrists, and occupational therapists. The total population consisted of 839 HCWs, comprising 559 nurses, 157 medical doctors, and 123 allied health workers (dietitians, physiotherapists, speech therapists, audiologists, dentists, dental therapists, radiographers, social workers, podiatrists, and occupational therapists).

Exclusion criteria included HCWs who were on leave at the time of data collection and those who were pregnant. Pregnant women were excluded to avoid potential bias, as physiological changes during pregnancy may influence eating habits and responses.

### 2.4. Sample Selection

A proportionate stratified random sampling technique was used to ensure representation of different professional categories within the hospital. The study population comprised nurses, medical doctors, and allied health professionals working across various departments of the tertiary hospital. A staff list obtained from the hospital’s Human Resources (HR) department served as the sample frame. The total number of participants in each stratum was determined proportionally to the size of that professional group within the hospital workforce. Within each stratum, participants were randomly selected using a random number generator and subsequently invited to participate in the study. This approach ensured that all professional categories were represented fairly in the sample. Although a stratified random sampling approach was used, participation was voluntary. Therefore, potential selection bias due to nonresponse cannot be completely excluded. Furthermore, staff availability during data collection may have influenced participation rates across professional categories.

The total number of healthcare workers employed at the tertiary hospital was 839, comprising 559 nurses, 157 medical doctors, and 123 allied health professionals. The minimum required sample size was determined using the Raosoft online sample size calculator. The parameters entered were a population size of 839, a confidence level of 95%, a margin of error of 5%, and an expected response distribution of 50%. The sample size was calculated using the primary study indicator, nutrition knowledge. Due to the absence of prior data on the prevalence of adequate nutrition knowledge among healthcare workers in this setting, a conservative expected proportion of 50% was used to maximise the required sample size. These inputs yielded a minimum sample size of 264 participants. To ensure representativeness across professional categories, a proportionate stratified sampling approach was used. The sample size for each stratum was calculated using the formula:

$$\mathrm{Sample}\;\mathrm{size}\;\mathrm{of}\;\mathrm{the}\;\mathrm{stratum}=(\frac{\mathrm{Total}\;\mathrm{sample}\;\mathrm{size}}{\mathrm{Population}\;\mathrm{size}})\times \mathrm{Stratum}\;\mathrm{size}$$

Applying this formula, the following sample sizes were derived:

- Nurses: (264 ÷ 839) × 559 = 176
- Medical doctors: (264 ÷ 839) × 157 = 49
- Allied health professionals: (264 ÷ 839) × 123 = 39

A total of 303 participants were ultimately recruited to improve the precision of estimates and account for potential non-response or incomplete data.

### 2.5. Data Collection

Participants were recruited through posters placed in hospital wards and through personal visits by the researcher. The poster included information on the study title, objectives, eligibility criteria, researcher contact details, and participation procedures. Prior to recruitment, permission was obtained from hospital management. Data were collected using a structured, self-administered questionnaire adapted from a previously validated instrument [20].

The questionnaire used in this study was adapted from a data collection tool which assessed dietary habits and eating practices among adolescents in rural and urban South African settings [20]. To ensure contextual relevance for the present study population, comprising adult healthcare workers in a tertiary hospital, the instrument was modified as follows: Terminology adjustments: Adolescent-specific wording was replaced with adult-appropriate language (e.g., “family meals” was changed to “main meals at home,” and “school tuck shop purchases” was changed to “canteen or vending machine purchases”). Contextual changes: Questions referring to school and community environments were revised to reflect workplace and hospital environments. Content modifications: Items focusing on adolescent behaviour (e.g., television watching, parental influence) were removed. New items assessing healthcare workers’ meal timing, shift-related eating patterns, and consumption of beverages (including energy drinks) were added.

Content validity was established through review by three experts in Human Nutrition and Public Health from Sefako Makgatho Health Sciences University. They evaluated each item for clarity, cultural appropriateness, and relevance to adult healthcare workers, leading to minor refinements in wording and layout.

Reliability testing was conducted in a pilot study among 30 healthcare workers from a non-participating hospital. Internal consistency of the adapted instrument was evaluated using Cronbach’s alpha, which showed good reliability (α = 0.70–0.80 for the nutrition knowledge section and α = 0.75–0.90 for the eating habits section). Based on the pilot findings, no substantial revisions were required before the main data collection.

While the questionnaire was adapted from a previously validated adolescent tool, the process included rigorous content validation and pilot testing to ensure suitability for an adult professional population. Nonetheless, as the instrument was newly adapted, further validation in similar populations is recommended for future studies.

The questionnaire, written in English, consisted of three sections: Section A collected socio-demographic information; Section B assessed nutrition knowledge; and Section C focused on eating habits. The self-administered format helped maintain participant anonymity, encouraging honest and accurate responses. The participants completed questionnaires during their breaks and within approximately 30 min.

Nutrition knowledge was assessed using 20 multiple-choice questions, with each correct response awarded one point. Based on their total scores, participants were classified into three categories: those scoring between 80 and 100 percent (16–20 points) were considered to have high nutrition knowledge, those scoring between 60 and 79 percent (12–15 points) were classified as having moderate knowledge, and scores below 60 percent (<12 points) were considered indicative of low nutrition knowledge.

Eating habits were assessed using 30 questions, of which 16 were used for scoring. Participants received one point for each “healthy” response. Those who scored between 80 and 100 percent (13–16 points) were classified as having healthy eating habits, while those who scored below 60 to 79 percent (<10–12 points) were considered to have unhealthy eating habits. The classification of scores for both nutrition knowledge and eating habits followed Bloom’s taxonomy cut-off criteria [21].

Data collection occurred over approximately three weeks, from 18 April to 5 May 2023, including weekdays and weekends. Questionnaires were completed primarily during morning staff meetings when staff were most accessible. Written informed consent was obtained from all participants and a participant’s information sheet was provided before they completed the questionnaire. The consent form with details of the researcher and signatures was attached. The researcher reviewed each questionnaire for completeness at the time of submission. To ensure confidentiality, the questionnaire did not contain any personal information about participants, such as names and identification numbers, nor did it contain employee numbers. The incomplete questionnaires were discarded. All completed forms were coded, stored securely in a box, and later entered into Microsoft Excel for data management.

A pilot study was conducted with 30 HCWs (10% of the sample size) to test the questionnaire’s clarity, structure, and content validity. Data from the pilot study were excluded from the main analysis. Feedback from the pilot was used to refine the wording, format, and instructions of the questions, ensuring the tool effectively addressed the study objectives.

### 2.6. Data Analysis

Data were analysed using IBM SPSS Statistics version 28. Descriptive statistics were used to summarise categorical variables (e.g., sex, profession) as frequencies and percentages. Inferential statistics were analysed using Pearson’s Chi-squared test to assess relationships between socio-demographic characteristics, nutrition knowledge, and eating habits. In this analysis, eating habits were treated as the dependent variable, while socio-demographic factors such as age were treated as independent variables. A *p*-value of <0.05 was considered statistically significant.

### 2.7. Ethical Considerations

All the respondents gave their informed consent for inclusion before they participated in the study. The study was conducted as per the Declaration of Helsinki, and the protocol was approved by Sefako Makgatho Health Sciences University Research Ethics Committee (SMUREC/H/344/2022: PG).

## 3. Results

### 3.1. Sociodemographic of Participants

The study sample that consented and completed the questionnaires comprised 303 participants. Most of the participants were nurses (55.8%), most of whom reported working in shifts. In terms of gender distribution, 66% were females and 34% were males. The age distribution revealed that most of the participants, 44.88% were between 22 and 35 years, and 7.59% were older than 55 years. Participants also had varied lengths of work experience: 56.1% had been employed for more than five years, 18.5% had 4–5 years of experience, 16.5% had 0–11 months, and 8.9% had 1–3 years, the lowest among the categories. Concerning work schedules, 54.1% reported working shifts, while 45.9% did not (Table 1).

### 3.2. Nutrition Knowledge of Healthcare Workers

Table 2 shows the responses of participants to the question of nutrition knowledge. The results show that most HCWs (84%) have sufficient knowledge of nutrition. Specifically, 55% of participants achieved a moderate level of knowledge (range from 60 to 79%), and 37% achieved a high level of knowledge (range from 80 to 100%). A small proportion (8%) had low nutritional knowledge. In the professional groups, doctors (19.5%) were recognised as having the highest level of nutrition knowledge compared to other health professionals.

### 3.3. Eating Habits

Table 3 presents the eating habits of the HCWs (n = 303). Most (81%) consumed 2–3 meals per day, while only 10% reported eating one meal per day, and a small proportion (9%) ate more than three meals per day. Similarly, 57% consumed one snack per day, while 40% had 2 to 3 snacks daily. Regarding meal patterns, 59% of participants reported eating breakfast, while 41% skipped it. Lunch intake was similar, with 59.5% eating lunch compared to 40.5% who did not.

In terms of food groups, most participants reported consuming vegetables (92%) and fruits (94%). Regarding the frequency of vegetable intake, 35% consumed vegetables twice a week, 30% consumed them daily, 28% consumed them 3–4 times a week, and only 7% consumed them five times a week. Similarly, for fruit intake, 43% reported daily consumption, 31% consumed fruits twice a week, 22% consumed them 3–4 times a week, and 4% consumed them five times a week.

A high proportion of participants engaged in less healthy practices: 80% ate chicken with skin, and 59% ate meat with skin. The majority (71%) of the participants consumed takeaways or fast food at least 3 to 4 times a week. A small percentage (8%) of participants did not buy takeaways or fast food, while 7% consumed takeaways daily and 14% consumed takeaways occasionally. In terms of preferred cooking methods, almost half (47%) preferred boiling and steaming, 22% used a variety of cooking methods, 18% grilled or baked, while 13% relied on frying or sautéing. When asked about preferred beverages, 60% chose sugary drinks, 17% preferred water, 12% opted for caffeinated drinks, 5% selected alcohol, and 7% reported consuming more than one type of drink. Less than half (45.5%) reported alcohol consumption, while 54.5% did not drink alcohol.

Overall, the summary of eating habits shows that only 16% of participants practised healthy eating patterns, while the majority (84%) followed unhealthy eating patterns.

### 3.4. Relationship Between Sociodemographic Characteristics and Nutrition Knowledge

Table 4 summarises the relationship between the socio-demographic variables and nutrition knowledge level of HCWs. The results indicate that there was no statistically significant relationship between nutrition knowledge and gender (*p* > 0.05). Similarly, no significant association was found between age and nutrition knowledge, although participants younger than 35 years had higher levels of nutrition knowledge compared to older age groups. A statistically significant association was observed between occupation and nutrition knowledge (*p* < 0.05), with nurses demonstrating a higher level of nutrition knowledge than both doctors and allied healthcare workers. There was no significant relationship between years of work experience and nutrition knowledge (*p* > 0.05). However, a significant difference was found concerning shift work: participants who worked in shifts exhibited better nutrition knowledge compared to those who did not work in shifts (*p* < 0.05).

### 3.5. Relationship Between Eating Habits and Socio-Demographics

Table 5 shows that there was a statistically significant relationship between age and eating habits among HCWs (*p* < 0.05). HCWs 35 years and younger were more likely to exhibit unhealthy eating habits compared to those older than 35 years. A significant association was also observed between occupation and eating habits, with nurses reporting a higher prevalence of unhealthy eating patterns compared to allied healthcare workers and medical doctors. Furthermore, eating habits were significantly correlated with years of work experience; healthcare workers who had more than five years of experience showed poorer dietary practices compared to those who had less than 5 years.

### 3.6. Relationship Between Nutrition Knowledge and Eating Habits of Healthcare Workers

Table 6 presents the relationship between nutritional knowledge and eating habits among HCWs. Although a greater proportion of participants across all levels of nutrition knowledge reported unhealthy eating habits, the relationship was not statistically significant (*p* = 0.389). Among those with low nutrition knowledge, 6.3% had unhealthy eating habits, while only 1.9% had healthy eating habits. Similarly, 45.5% of participants with moderate nutrition knowledge reported unhealthy eating habits, compared to 9% with healthy habits. Even among those with high nutrition knowledge, 32.3% still exhibited unhealthy eating habits, with only 5% practicing healthy eating.

### 3.7. Occupation and Source of Lunch

Table 7 shows that the majority of the HCWs who do not carry a lunchbox to work rely on fast food and takeaways as their source of lunch. Their findings were significant with a *p* <0.05

## 4. Discussion

The study aimed to evaluate the knowledge and food habits of health workers (HCWs) at a tertiary hospital in the Limpopo province of South Africa. The findings of the study showed that employment influences the eating habits of participants. Although the results of the study indicated that nurses had higher levels of nutrition knowledge than allied health professionals and medical professionals, these findings could have been influenced by the population of the study (most participants were nurses). According to the South African Health Professionals Council (2025), dietitians are health professionals with the highest level of nutrition knowledge because their training is specialised in nutrition and nutritional therapy. The apparent discrepancy in this study is probably due to methodological limitations. Specifically, the number of dietitians participating was very low compared to the number of nurses, which may have distorted the findings and underestimated the level of dietitian knowledge. A larger and more balanced sample of the professional group would provide a more accurate reflection of the relative level of nutrition knowledge.

Despite nurses having moderate (32.8%) to high (16.8) levels of nutrition knowledge, almost half of the nursing staff (45.2%) practice unhealthy eating habits. These included frequent consumption of sugary foods and beverages, fatty foods, and fast food, as well as meal skipping and low intake of fruits and vegetables. Studies have indicated that prolonged working hours can greatly contribute to the development of unhealthy lifestyles among employees. Such negative outcomes include a higher tendency to engage in behaviours like poor nutrition (reliance on fast foods and processed foods), smoking, physical inactivity, insufficient sleep, and excessive alcohol intake. Moreover, extended work hours are linked to elevated levels of work-related stress and reduced leisure time, which increase the likelihood of substance [21]. The availability and accessibility of sugary, fatty, and healthy foods sold by vendors surrounding the hospital influence the likelihood of healthcare workers consuming these types of food. Dietitians are encouraged to collaborate with food vendors to increase the availability of healthier options, providing recognition or incentives to vendors who sell nutritious meals. Additionally, dietitians should work with hospital management to develop policies that regulate the foods sold by vendors in and around the hospital.

More than half of the participants reported consuming breakfast daily. However, breakfast options varied, with cereals and milk being the most consumed, while some participants reported eating leftovers or confectionery. Breakfast should ideally be consumed within two hours of waking, before 10 a.m., and is considered the most important meal of the day [22]. Skipping breakfast has been linked to increased risk of overweight and obesity, primarily due to overconsumption during subsequent meals [22]. The Food-Based Dietary Guidelines for South Africa (FBDG-SA) [23] recommend consuming five to six meals per day, including two to three main meals and two to three snacks. However, most HCWs in this study reported consuming only two to three meals per day, with a minority consuming more than five meals. Additionally, most participants consumed only one snack per day. This suggests poor adherence to dietary guidelines, which may compromise nutritional adequacy, especially in terms of vitamins and minerals important for immune function [24]. Time constraints and demanding schedules likely contributed to these patterns [25].

Although most HCWs indicated a preference for home-cooked meals, many still consumed fast foods three to four times a week. Similar findings were reported by [26] who found that time limitations attributed to the long hours of work that they are faced with often prevented HCWs from preparing meals at home, leading them to rely on takeaways. Frequent consumption of fast foods is considered unhealthy due to their high energy density and low nutrient content, and because they are often served in large portion sizes [27]. There is a clear need to create healthier food environments within healthcare settings and involve dietitians in policy development to ensure that most foods in the cafeteria are healthy.

The study found that most HCWs preferred sugary beverages such as sodas or soft drinks, which are rich in simple sugars and have been linked to obesity, type 2 diabetes, hypertension, and certain cancers [28]. These beverages are often consumed as a coping mechanism for work-related stress and fatigue [29]. Only a few participants reported drinking water regularly. According to the FBDG-SA, adults should consume at least eight glasses of water daily. Water plays a critical role in physiological processes, including digestion, and constitutes approximately 70% of the human body [30]. Increasing water intake among HCWs should be prioritised, and they should be encouraged to carry water bottles to work to promote regular hydration.

Daily consumption of fruits and vegetables was low among participants, despite guidelines recommending their frequent intake for disease prevention and overall health [23]. This is concerning, as fruits and vegetables are vital sources of dietary fibre, antioxidants, and micronutrients that support immune function. To address this, HCWs should be encouraged to grow home gardens and to incorporate raw fruits and vegetables into their meals, particularly in lunchboxes, to improve accessibility and intake.

It is important to note that the study did not find any influence of nutrition knowledge on eating habits (*p* > 0.05). Even participants with high nutrition knowledge are often involved in unhealthy food habits. This is consistent with the conclusions that knowledge alone does not necessarily lead to healthy eating practices [31]. In various occupational groups, there was no influence on knowledge and eating habits. Most of the nurses had higher nutrition knowledge, but many still reported bad eating habits. A similar trend was observed among doctors and allied health workers in India. [32].

The world-systems theory largely contributes to the way people (including HCWs) eat daily. Factors such as trade liberalisation increase the importation of food, which increases access to highly processed foods that are high in sugar and unhealthy fats. Cultural globalisation increases the appeal of Western foods over traditional foods. This could be one of the factors why people continue to consume unhealthy foods despite knowing [33]. Furthermore, modernisation theory has also been identified as a factor that contributes to unhealthy eating due to domestic factors. This theory believes that economic growth increases urbanisation and increases the rate of employment among women. Culturally, women are expected to stay at home and prepare meals for their families, but because of this transition, they opt for convenient and affordable meals, which are ultra-processed (high in salt, fats, and sugars) [33].

The findings are concerning, as HCWs are expected to be role models for healthy habits. Their poor diets may undermine their credibility when educating patients or the public. If HCWs continue to adopt unhealthy diets, this could undermine efforts to promote healthy lifestyles and reduce the burden of disease in a wider population.

There is a critical need for targeted interventions to address the barriers to HCWs making healthy dietary choices. Effective strategies can include the implementation of workplace wellness programmes that actively promote healthy eating, as well as practical training in meal planning and food preparation. The revision of hospital food service policies to ensure greater access to nutritious meals is also essential, while encouraging nurses to carry lunch boxes and water bottles may help to promote healthier daily habits. Dietitians must play a central role in the development and implementation of cafeteria and food vendor guidelines to ensure consistent availability of healthy food options in healthcare facilities.

## 5. Study Limitations

The study was conducted at a single tertiary hospital in the Limpopo province of South Africa. The findings may not be generalisable to all healthcare workers in the province or across South Africa, given the limited geographic and institutional scope of the study. The research methodology applied led to a high sample of nurses compared to the medical doctors and allied workers, which was influenced by the total population size.

## 6. Conclusions

This study found no relationship between nutrition knowledge and eating habits, indicating that knowledge alone does not guarantee healthy eating. Although they have sufficient knowledge of nutrition, many health workers continue to follow unhealthy eating habits. Further research is recommended to explore the barriers to healthcare workers following a healthy diet and assess the hospital food environment, including the availability of nutritious alternatives. Food vendors within the health environment should be monitored and encouraged to provide healthier options while limiting foods rich in saturated fats and sugar.

## Figures and Tables

**Table 1:** Sociodemographic data of healthcare workers (n = 303).

Variable	n (%)
Gender	
Male	102 (34.0%)
Female	201 (66.0%)
Population group	
African	301 (99.3%)
Indian	2 (0.7%)
Age group	
22–35 years	136 (44.88%)
36–45 years	85 (28.05%)
46–55 years	59 (19.47%)
>55 years	23 (7.59%)
Mean age (32.78)	
Median age (36)	
Occupation	
Nursing	169 (55.8%)
Medical doctors	59 (19.5%)
Allied	75 (24.7%)
Work experience	
0–11 months	50 (16.5%)
1–3 years	27 (8.9%)
4–5 years	56 (18.5%)
>5 years	170 (56.1%)
Shift worker	
No	139 (45.9%)
Yes	164 (54.1%)

**Table 2:** Nutrition knowledge response by healthcare workers.

Variable	Correct	Incorrect	I Don’t Know
	n (%)	n (%)	n (%)
A balanced diet is a diet that includes the consumption of water and fruits.	84 (27.7%)	215 (71%)	4 (1.3%)
A balanced diet is a diet that includes a variety of foods in adequate amounts.	256 (84.2%)	41 (13.5%)	7 (2.3%)
Lettuce, spinach are good sources of protein	156 (51.5%)	142 (46.9%)	5 (1.7%)
Bread, rice, cereal and pasta are good sources of CHO	270 (89.1%)	30 (9.9%)	3 (1%)
Citrus fruits are good sources of vitamin C	289 (95.4%)	10 (3.3%)	4 (1.3%)
Daily recommended intake of fruits and vegetables is 3–5	233 (76.9%)	65 (21.5%)	5 (1.7%)
Daily recommended intake of fruits and vegetables is 5–6	186 (61.4%)	110 (36.3%)	7 (2.3%)
CHO is our body’s best source of energy	244 (80.4%)	54 (17.8%)	5 (1.7%)
Milk and dairy products are good sources of calcium	284 (93.77%)	19 (6.3%)	0 (0%)
Chocolates, sweets and chips are healthy snacks	274 (90.4%)	28 (9.2%)	1 (0.3%)
Omitting CHO is healthy	229 (75.6%)	72 (23.8%)	2 (0.7%)
Most processed foods are high in sugar, salt and fats	269 (88.8%)	32 (10.6%)	2 (0.7%)
Mono and polyunsaturated fats are healthy	152 (50.2%)	143 (47.2%)	8 (2.6%)
Peanuts are a good source of zinc	136 (44.9%)	160 (52.8%)	7 (2.3%)
Skipping meals is healthy	253 (83.5%)	49 (16.2%)	1 (0.3%)
Iron sources are from animal products and fish	214 (70.6%)	85 (28.4%)	3 (1%)
Avocados are a healthy fat	281 (92.7%)	21 (6.9%)	1 (0.3%)
Whole Grains are a source of food	285 (94.1%)	15 (5%)	3 (1%)
Vegetable oils raise blood cholesterol	97 (32%)	201 (66.3%)	5 (1.7%)
Recommended intake is 6–8 glasses	286 (94.4%)	17 (5.6%)	0 (0%)

**Table 3:** Eating habits of healthcare workers.

Variable		Frequency (n)	Percentage %
Number of meals consumed per day	1 meal	30	10
	2–3 meals	245	81
	4–5 meals	21	7
	>5 meals	6	2
Number of snacks consumed per day	1 snack	173	57
	2–3 snacks	121	40
	4–5 snacks	6	2
	>5 snacks	3	1
Breakfast intake	Yes	179	59
	No	124	41
Lunch intake	Yes	180	60
	No	123	40
Vegetable intake	Yes	279	92
	No	24	8
Fruits intake	Yes	285	94
	No	18	6
Eat chicken with skin	Yes	242	80
	No	61	20
Eat meat with skin	Yes	129	59
	No	124	41
Drink alcohol	Yes	138	46
	No	165	54
Consumption of take aways	Daily	21	7
	3–4 times a week	215	71
	Occasionally/monthly	43	14
	Do not buy	24	8
Preferred beverages	Sugary drinks	182	60
	Alcohol	15	5
	Water	52	17
	Caffeinated drink	35	12
	>1 drink type	19	6
Preferred cooking methods	Frying and sauteing	39	13
	Boiling and steaming	142	47
	Grilling and baking	55	18
	Variety	67	22
Frequency of vegetable intake	Daily	91	30
	2 times a week	106	35
	3–4 times a week	85	28
	5 times a week	21	7
Frequency of fruit intake	Daily	130	43
	2 times a week	94	31
	3–4 times a week	67	22
	5 times a week	12	4
Summary of eating habits	Healthy eating	48	16
	Unhealthy eating	255	84

**Table 4:** Relationship between sociodemographic characteristics and nutrition knowledge.

Nutritional Knowledge of Healthcare Workers				
	Low (<50% Score)	Moderate (60–79% Score)	High (80–100% Score)	*p*-Value
Gender				
Male	8 (2.6%)	56 (18.5%)	38 (12.5%)	0.982
Female	17 (5.6%)	109 (36%)	75 (24.8%)	
Age				
22–35	5 (1.7%)	73 (24%)	58 (19.1%)	0.116
36–45	8 (2.6%)	49 (16.2%)	28 (9.2%)	
46–55	8 (2.6%)	32 (10.6%)	19 (6.3%)	
56+	4 (1.3%)	11 (3.6%)	8 (2.6%)	
Occupation				
Allied	5 (1.7%)	38 (12.5%)	32 (10.6%)	0.006 *
Medical	0 (0.00%)	29 (9.6%)	30 (9.9%)	
Nursing	20 (6.6%)	98 (32.3%)	51 (16.8%)	
Experience				
0–1	1 (0.33%)	27 (8.9%)	22 (7.3%)	0.159
2–3	1 (0.3%)	15 (5%)	11 (3.6%)	
4–5	2 (0.66%)	33 (10.9%)	21 (6.9%)	
5+	21 (6.9%)	90 (29.7%)	59 (16.8%)	
Working in shifts				
No	5 (1.7%)	69 (22.8%)	65 (21.5%)	0.001 *
Yes	20 (6.6%)	96 (31.7%)	48 (15.8%)	

* *p*-value ≤ 0.05 statistically significant.

**Table 5:** Relationship between eating habits and socio-demographics.

Sociodemographic Characteristics	Eating Habits		*p*-Value
	Healthy (80–100% Score)	Unhealthy (60–79% Score)	
Age			
22–35	8 (2.6%)	128 (42.2%)	0.000 *
36–45	15 (5%)	70 (23%)	
46–55	8 (2.6%)	51 (16.8%)	
56+	7 (2.3%)	16 (5.3%)	
Profession			
Allied	14 (4.6%)	61 (20.1%)	0.014 *
Medical doctors	2 (0.6%)	57 (18.8%)	
Nursing	32 (10.6%)	137 (45.2%)	
Experience			
0–1	5 (1.7%)	45 (14.9%)	0.015 *
2–3	0 (0.0%)	27 (8.9%)	
4–5	7 (2.3%)	49 (16.2%)	
5+	36 (11.9%)	134 (44.2%)	
Shift worker			
No	14 (4.6%)	125 (4.3%)	0.011 *
Yes	34 (11.2%)	130 (42.9%)	

* *p*-value ≤ 0.05 statistically significant.

**Table 6:** Relationship between nutrition knowledge and eating habits of healthcare workers.

Eating Habits	Nutritional Knowledge				*p*-Value
	Low	Moderate	High	Total	
Healthy habits	6 (1.9%)	27 (8.9%)	15 (4.9%)	48 (15.84%)	0.389 **
Unhealthy habits	19 (6.3%)	138 (45.5%)	98 (32.3%)	255 (84.16%)	
Total	25 (8.3%)	165 (54.5%)	113 (37.3%)	303 (100%)	

** *p*-value ≥ 0.05 not statistically significant.

**Table 7:** Cross tabulation between occupation and their source of lunch.

Occupation	Source of Lunch		*p*-Value
	Fast Foods and Takeaways	Do Not Eat Lunch (Skip Lunch)	
Nursing	32 (35.2%)	4 (12.5%	0.030 *
Medical	29 (63%)	17 (37%)	
Allied	30 (73.2%)	11 (26.8%)	

* *p*-value ≤ 0.05 statistically significant.

## Data Availability

The data presented in this study are available upon request from the corresponding author.
